# A critical analysis of CellaVision systems in the modern hematology laboratory

**DOI:** 10.1093/ajcp/aqaf045

**Published:** 2025-05-25

**Authors:** Brydon Panozzo, Jaineel Ramnarain, Song Chen, Hiu Lam Agnes Yuen, Maciej Tatarczuch, Shahla Vilcassim, Christopher Chang-Yew Leow, Chris Barnes

**Affiliations:** Sir Peter MacCallum Department of Oncology, The University of Melbourne, Melbourne, Victoria, Australia; Australian Clinical Labs, Melbourne, Victoria, Australia; Australian Clinical Labs, Melbourne, Victoria, Australia; Australian Clinical Labs, Melbourne, Victoria, Australia; Australian Clinical Labs, Melbourne, Victoria, Australia; School of Clinical Sciences, Monash University, Melbourne, Victoria, Australia; Australian Clinical Labs, Melbourne, Victoria, Australia; Department of Haematology, Alfred Health, Melbourne, Victoria, Australia; School of Clinical Sciences, Monash University, Melbourne, Victoria, Australia; Australian Clinical Labs, Melbourne, Victoria, Australia; School of Clinical Sciences, Monash University, Melbourne, Victoria, Australia; Australian Clinical Labs, Melbourne, Victoria, Australia; Australian Clinical Labs, Melbourne, Victoria, Australia

**Keywords:** CellaVision, telepathology, digital morphology

## Abstract

**Objective:**

This review aims to provide a comprehensive analysis of the current literature regarding the use of CellaVision digital morphology systems and to assess the emerging applications, their potential to supplement current diagnostic pathways, and—importantly—appreciate their practical and perceived limitations.

**Methods:**

A manual literature review was conducted to identify relevant journal articles and published abstracts as they relate to the application of CellaVision systems to hematology laboratory practice in human patients.

**Results:**

CellaVision systems can characterize cellular morphology with overall a high degree of accuracy—in particular, for neutrophils; lymphocytes; and common red blood cell changes, including target cells. Challenges remain, however, with the detection of particular important findings, including immature granulocytes and red blood cell agglutination. The application of CellaVision systems to emerging areas such as telepathology and parasitology are evolving, with an increasing volume of literature highlighting the technology’s utility.

**Conclusion:**

CellaVision systems and associated digital technologies are poised to play an increasingly important role in hematology laboratories, with promises of enhanced accuracy and improved workflows. Several critical deficiencies highlight the necessity for continued research to support their transition into routine clinical practice.

**KEY POINTS**

- The CellaVision system can characterize common cellular morphology with an overall high degree of accuracy.
- Critical blood film abnormalities, including the detection of platelet clumping and blast cells, remain an important challenge that the CellaVision system has yet to overcome.
- Oversight by skilled laboratory staff remains key to the successful implementation and use of digital morphology systems.

## INTRODUCTION

Blood film analysis has continued to evolve ever since the first red blood cells (RBCs) were described by Dutch physician Antoni van Leeuwenhoek in 1674. The use of manual light microscopy for the examination of Romanowsky-stained peripheral blood smears has formed an essential part of the diagnosis of hematologic and nonhematologic diseases for more than 3 centuries.^1,2^ These methods have not been without their disadvantages, with considerable rates of interobserver variability and a requirement to continuously train and accredit skilled and technically competent staff.^3^

Digital microscopy systems involve the use of digital photography and, increasingly commonly, computer-assisted analysis for the morphologic evaluation of slides. They have been developed with the promise that they offer an alternative to conventional manual light microscopy within the hematology laboratory. The proposed benefits of their implementation have included enabling digital archiving of images, enabling the remote review of blood film slides, reducing the physical occupational health and safety hazards of conventional microscopy, and potentially facilitating higher throughput and reduced labor costs.^4^

Four such commercially available systems that are available as stand-alone units are the CellaVision DC-1 analyzer, the CellaVision DM96 automated image analysis system, the CellaVision DM1200 digital cell morphology system, and the CellaVision DM9600 digital cell morphology analyzer (CellaVision AB). These systems have the capacity to identify items of interest within a slide (ie, blood cells), digitally photograph each cell, preclassify the cell using an artificial neural network, then transmit and display the image for further analysis and storage.^5^ The CellaVision DC-1 analyzer loads a single slide and is designed for low-throughput workflows. The CellaVision DM96 system (the precursor to the CellaVision DM9600 analyzer) is the system most widely reported on in the literature and consists of a slide feeder, a 3-objective (×10, ×50, and ×100) microscope, a digital camera, and a computer system. The system is capable of scanning up to 30 slides per hour at ×10 or 2 slides per hour at ×10 and ×50.^6^

Here, we examine the available data on the application of CellaVision systems within the hematology laboratory. Despite the enticing potential of improved accuracy and cost-effectiveness, a transition away from traditional workflows requires a well-validated and robust evidence base to be established before the technology can see widespread adoption.

## METHODS

A literature search was conducted of the electronic databases PubMed and Medline. The search included all articles published until October 2024. Stand-alone search terms used included “CellaVision” and “digital morphology” and returned 111 unique results (209 duplicates). Relevant articles were identified through a manual screen of titles and abstracts and limited to studies written in English. An additional search was conducted on the CellaVision website and of the websites of related professional interest groups.

## CORE FUNCTIONS OF CELLAVISION SYSTEMS

### Differential white blood cell count

One of the core functions of the CellaVision systems is the ability to perform an automated differential count, or “preclassification.” Images of each cell and the cell’s classification are then reviewed by a morphologist and reclassified if adjustments are required before result validation. Several studies have attempted to quantify the preclassification accuracy of the DM96 system, with variable results [Table 1]. Rollins-Raval et al^8^ examined patient samples from the adult general hospital, with the greatest preclassification accuracy demonstrated in neutrophils (96.46%) and lymphocytes (95.96%) and the lowest accuracy in eosinophils (65.53%) and basophils (62.11%). This finding of greater accuracy in the preclassification of neutrophils and lymphocytes than in the preclassification of eosinophils and basophils is largely consistent with early findings in 3 separate studies.^5,9,10^ When implemented across multiple sites within a single hospital network, the 5-part white blood cell (WBC) differential count demonstrated minimal interlaboratory variation.^11^ Stouten et al^7^ conducted the largest study to date to review and compare the preclassification accuracy of 6945 slides to expert manual assessment. The authors demonstrated a high degree of correlation (*R* > 0.9) across all 5 major WBC types. The authors noted that the CellaVision software did not possess the capacity to individually classify certain WBC types deemed clinically relevant (eg, promonocytes, prolymphocytes, hairy cells) and demonstrated inferior correlation in the preclassification accuracy of less common WBC types (eg, plasma cells [*r*^2^ = 0.576]).

**Table 1:** Summary of Studies Examining the WBC Differential of Various CellaVision Systems’ Preclassified Results Against a Reference Method of Manual Microscopy Alone or Reclassification^a^ of Digital Microscopy Images

Author (year)	System	Blood smears, No.	Correct suggestion, %	Correlation coefficient, *R*	95% CI	Correct suggestion, %	Correlation coefficient, *R*	95% CI	Correct suggestion, %	Correlation coefficient, *R*	95% CI	Correct suggestion, %	Correlation coefficient, *R*	95% CI	Correct suggestion, %	Correlation coefficient, *R*	95% CI
			Neutrophils			Lymphocytes			Eosinophils			Monocytes			Basophils		
Stouten et al (2015)^7^	DM96	6945	N/A	0.997	0.99-0.99	N/A	0.997	0.99-0.99	N/A	0.978	0.90-0.92	N/A	0.933	0.90-0.99	N/A	0.928	0.80-0.89
Rollins-Raval et al (2012)^8^^a^	DM96	51699^b^	96.46	N/A	N/A	95.96	N/A	N/A	65.53	N/A	N/A	88.7	N/A	N/A	62.11	N/A	N/A
Briggs et al (2009)^9^	DM96	136	99.5	0.95	Not stated	94.9	0.73	Not stated	79.9	0.5	Not stated	87.6	0.12	Not stated	54.1	0	Not stated
Ceelie et al (2007)^10^	DM96	200	98.6	N/A	N/A	95.2	N/A	N/A	93.5	N/A	N/A	94	N/A	N/A	84.7	N/A	N/A
Kratz et al (2005)^5^	DM96	120	92.5	N/A	N/A	96.4	N/A	N/A	63.2	N/A	N/A	81.4	N/A	N/A	80.0	N/A	N/A

Abbreviations: DM96, CellaVision DM96 automated image analysis system; N/A, not applicable; WBC, white blood cell.

^a^ Reclassified image.

^b^Events counted—stated as approximately 100 events per slide.

Five major studies to date have examined the correlation between reclassified DM96 system results and manual microscopy [Table 2]. Lee et al^12^ published the largest and most recent study to date examining 359 slides from patients at 3 adult and 1 pediatric hospital in addition to 1 community laboratory. Correlation was highest for neutrophils (*r*^2^ = 0.96) and lymphocytes (*r*^2^ = 0.95) and lowest for monocytes (*r*^2^ = 0.88), eosinophils (*r*^2^ = 0.83), and basophils (*r*^2^ = 0.76). These findings are similar to those published by Park et al^13^ from the University of Ulsan College of Medicine and Asan Medical Center, Seoul, Korea, the previous year. It is important to note when interpreting the correlation between the DM96 system and the manual differential count that considerable variance exists between manual differential counts and a reference method. Koepke et al^14^ compared the differential counts of 73 technologists across 5 laboratories with an expert reference. Correlation was highly variable between the cell types, being highest for neutrophils (*r*^2^ = 0.87) and eosinophils (*r*^2^ = 0.83) and considerably lower for lymphocytes (*r*^2^ = 0.73), monocytes (*r*^2^ = 0.41), and basophils (*r*^2^ = 0.32). With respect to the performance of the CellaVision system in the classification of basophils specifically, the 2 earliest works by Kratz et al^5^ and Ceelie et al^10^ did not publish correlative data, and Briggs et al^9^ alone published evidence of poor correlation with their reference method (*r*^2^ = 0.05). It could be reasonably hypothesized that the relatively low number of circulating basophils in the specimens and a degree of selection bias in the allocation of peripheral blood smears for analysis between studies and across sites may have contributed to the increased variation in basophil correlation to manual methods.

**Table 2:** Summary of Studies Examining the WBC Differential of Various CellaVision Systems’ Reclassified Results Against a Reference Method of Manual Microscopy

Author (year)	System	Blood smears, No.	Correlation coefficient, *R*	95% CI	Correlation coefficient, *R*	95% CI	Correlation coefficient, *R*	95% CI	Correlation coefficient, *R*	95% CI	Correlation coefficient, *R*	95% CI
			Neutrophils		Lymphocytes		Eosinophils		Monocytes		Basophils	
Lee et al (2013)^12^	DM96	359	0.96	Not stated	0.95	Not stated	0.88	Not stated	0.83	Not stated	0.76	Not stated
Park et al (2013)^13^	DM96	298	0.99	Not stated	0.99	Not stated	0.96	Not stated	0.94	Not stated	0.86	Not stated
Briggs et al (2009)^9^	DM96	136	0.99	Not stated	0.96	Not stated	0.67	Not stated	0.81	Not stated	0.05	Not stated
Ceelie et al (2007)^10^	DM96	136	0.95	Not stated	0.94	Not stated	0.85	Not stated	0.70	Not stated	N/A	N/A
Kratz et al (2005)^5^	DM96	120	0.96	Not stated	0.94	Not stated	0.73	Not stated	0.67	Not stated	N/A	N/A

Abbreviations: DM96, CellaVision DM96 automated image analysis system; N/A, not applicable; WBC, white blood cell.

The performance of the CellaVision DM96 system in completing accurate neutrophil counts in neutropenic patients has been examined by Amundsen et al^15^ at Oslo University Hospital. The researchers evaluated 106 patient samples with neutrophil counts below 2.0 × 10^9^/L using a flow cytometry reference method and the CellaVision DM96 system. The CellaVision system demonstrated excellent concordance with the reference method (*R*^2^ = 0.958).

The requirement for neutrophil reclassification before result validation has recently been challenged in a large Danish retrospective analysis by Christiansen et al^16^ that examined the preclassification and reclassification neutrophil counts of 4354 samples over a 4-year period. The authors reported a median difference in preclassificaiton and reclassified neutrophil counts of 0.044 × 10^9^/L (95% CI, 0.035-0.052 × 10^9^/L). The preclassification sensitivity and specificity for the detection of neutrophilia was 98.8% and 97.2%, respectively, and for neutropenia was 97.7% and 99.1%, respectively. The authors concluded that there was satisfactory diagnostic performance, including at relevant clinical decision levels. The use of preclassification neutrophil counts as a reportable “preliminary” result may facilitate a faster turnaround time to final result publication than current workflows.

The accurate and timely recognition of blast cells is an essential component of the peripheral blood film review. Cornet et al^17^ examined the performance of the DM96 system in a cohort of 84 patients with known malignant hematologic diseases. A specific analysis to assess the sensitivity of the system to detected blasts was completed on a subset of 34 patients with acute myeloid leukemia (AML), acute lymphoblastic leukemia/lymphoma (ALL), and myelodysplastic syndrome. The DM96 system appropriately detected blasts in 100% of the provided samples. With regard to blast counts compared with manual microscopy, the correlation with the DM96 system preclassification was moderate (*R*^2^ = 0.5716). Following reclassification, including cells the system identified as “unclassified,” the correlation improved substantially (*R*^2^ = 0.9). The DM96 system tended to underestimate the blast count, most notably in cases of ALL, with frequent misclassification of lymphoid blasts as lymphocytes. Acute promyelocytic leukemia (APL) is a subtype of AML, classified by a translocation between chromosomes 15 and 17 involving the *PML* and *RARA* genes and morphologically characterized by the accumulation of abnormal promyelocytes in the blood and bone marrow. Early identification and therapy are critical because the disorder is associated with a severe bleeding diathesis and early mortality.^18^ The CellaVision peripheral blood application system does not possess an individual preclassification category for abnormal promyelocytes or blasts containing Auer rods. Investigational work has been undertaken to explore how the further analysis of data captured by the CellaVision system can be used to predict the likelihood of APL using deep learning techniques. John-William Sidhom et al^19^ from Johns Hopkins University School of Medicine trained and validated deep learning models using the peripheral blood films of 106 AML cases (72 non-APL, 34 APL). Training the model using a multiple-instance learning approach yielded a receiver operating characteristic (ROC) area under the curve of 0.91 in the discovery cohort and 0.86 in the validation cohort when distinguishing APL from non-APL. The model’s performance was compared with the performance of 10 practicing academic physicians on the morphologic assessment of 12 APL and 12 non-APL AML cases demonstrated that the model performed with equivalent or better classification performance. The study also described a portion of the characteristics the model used to discriminate the cases, including the proportion of nonblast cell types (ie, erythroblasts, segmented neutrophils and platelets) and the nuclear pixel distribution of APL cells. Laura Boldú et al^20^ from Hospital Clínic de Barcelona-IDIBAPS has similarly demonstrated how deep learning techniques can be applied to images captured by the CellaVision system to predict leukemia lineage and assist in the determination of leukemia subtype.

The capacity of the CellaVision system to classify immature granulocytes has also been examined more broadly. Brigs et al^9^ demonstrated that although for immature granulocytes as a single category the agreement between the reference method and the reclassified DM96 system result was high (*r*^2^ = 0.95), for each individual stage of maturity the strength of the correlation was substantially lower (promyelocytes, *r*^2^ = 0.42; myelocytes, *r*^2^ = 0.37; and metamyelocytes, *r*^2^ = 0.93). The authors noted that the accurate classification of immature granulocytes is known to be prone to substantial variation when performed manually. This finding is highlighted by the substantial variation between 5 morphologists’ classification of each-stage maturity compared with a 400-cell reference standard: promyelocytes (*r*^2^ = 0.018-0.948), myelocytes (*r*^2^ = 0.264-0.804), and metamyelocytes (*r*^2^ = 0.274-0.863).

The morphologic assessment of pediatric blood films must account for children’s age-specific hematopoiesis, which necessitates special consideration of this patient cohort.^21^ Rollins‐Raval et al^8^ examined the preclassification performance of the CellaVision DM96 system in 8009 events at Children’s Hospital of Pittsburgh. Preclassification accuracy was highest for lymphocytes (99.47%), monocytes (99.47%), and neutrophils (97.24%) and was lowest for eosinophils (79.70%) and basophils (37.88%). The CellaVision preclassification performed well in blast cell identification, with a positive predictive value and sensitivity of 94.7% and 90.5%, respectively. In an earlier study, Billard et al^22^ examined the influence of age in a cohort of 277 pediatric patients without a hematologic disorder or severe infection. The overall postclassification agreement of the 5-part WBC differential was 87% but was lower for newborns and children 1 to 7 days of age, at 81% and 79.3%, respectively.

The potential ability of the CellaVision system to decrease the time required for the completion of a WBC differential and manual film review is an area of keen interest. Kratz et al^5^ performed 2 timing studies. The first compared the time taken for 6 technologists with extensive experience with manual microscopy to complete 10 manual differentials with either a manual microscope (average, 5.1 minutes) or the CellaVision DM96 system (average, 6.4 minutes). The authors acknowledged the limitations of this study, including the limited experience of the technologists with the CellaVision system. A subsequent study was conducted in which 2 participants with different levels of experience received training on the CellaVision DM96 system. In this study, the performance of the experienced technologist was similar across both the CellaVision and the manual microscope (1.5 minutes vs 1.4 minutes on average per slide), respectively, with the less experienced technologist being faster with the CellaVision system (3.0 minutes vs 4.1 minutes on average per slide). Furthermore, Brigs et al performed a timing study comparing the time taken for 5 scientists to complete 30 WBC differentials on the CellaVision system compared with manual microscopy, the authors found using the CellaVision system to be, on average, faster than manual microscopy, with no reduction in precision (80 minutes vs 174 minutes, respectively).^9^ Of note, no formal statistical analysis of the timing differences was reported.

### Assessment of RBC morphology

The CellaVision system can be used in conjunction with the CellaVision Advanced RBC Application software. This platform employs artificial intelligence (AI) to classify RBCs based on 21 morphologic characteristics broken down by color, size, shape, and the presence or absence of inclusions. Individual cutoffs and classification parameters are customizable by the individual laboratory, which can make directly comparing studies on the accuracy and precision of the software challenging.

Park et al^23^ from the Department of Laboratory Medicine, Korea University Guro Hospital, in Seoul examined the correlation of the CellaVision Advanced RBC Application software with respect to RBC characterization and grading against the 2015 International Council for Standardization in Haematology guidelines. A total of 223 samples (123 abnormal and 100 controls) were analyzed using the CellaVision DM9600 analyzer and manual microscopy. With respect to preclassification accuracy, the highest sensitivity was reported for target cells (100% [95% CI, 47.8%-100%]) and teardrop cells (100% [95% CI, 73.5%-100%]), and the lowest sensitivity was reported for acanthocytes (10% [95% CI, 0.3%-44.5%]) and spherocytes (11.1% [95% CI, 0.3%-48.3%]) [Table 3]. The preclassification severity grading agreement was highest for spherocytes and acanthocytes and lowest for echinocytes and schistocytes. These findings were similar to earlier work completed by Criel et al^24^ at the Department of Laboratory Hematology, AZ Sint-Jan, Bruges, Belgium. The team examined 106 samples and used a method of cutoff “optimization,” with the goal of achieving 80% or greater sensitivity for target cells, teardrop cells, spherocytes, and sickle cells and 80% or greater specificity for other categories. Employing cutoffs derived from the ROC curve of the entire lot of samples achieved 80% or greater sensitivity for target cells, teardrop cells, and sickle cells among others. The software remained particularly insensitive (≤50%) to microcytes, ovalocytes, and parasites.

**Table 3:** Summary of Studies Examining RBCs of Various CellaVision Systems’ Preclassified Results Against a Reference Method of Manual Microscopy

Author (year)	System	Blood smears, No.	Cutoff, %	Sensitivity, % (95% CI)	Specificity, % (95% CI)	Cutoff, %	Sensitivity, % (95% CI)	Specificity, % (95% CI)	Cutoff, %	Sensitivity, % (95% CI)	Specificity, % (95% CI)	Cutoff, %	Sensitivity, % (95% CI)	Specificity, % (95% CI)	Cutoff, %	Sensitivity, % (95% CI)	Specificity, % (95% CI)
			Acanthocytes			Echinocytes			Elliptocytes			Spherocytes			Target Cells		
Park et al (2021)^23^	DM9600	223	>5	10.0 (0.3-44.5)	98.1 (95.3-99.5)	>5	88.9 (51.8-99.7)	74.3 (67.9-80.0)	>5	83.3 (62.6-95.3)	86.4 (80.9-90.9)	>5	11.1 (0.3-48.3)	99.5 (97.4-100)	>5	100 (47.8-100)	96.3 (92.9-98.4)
Criel et al (2016)^24^	DM96	106	≥5	50 (N/S)	98 (N/S)	≥10	42.9 (N/S)	98 (N/S)	≥6	0 (N/S)	100 (N/S)	≥1	0 (N/S)	94.8 (N/S)	≥5	50 (N/S)	100 (N/S)
Horn et al (2015)^25^	DM96	198	>5	75 (N/S)	97 (N/S)	>10	47 (N/S)	96 (N/S)	N/A	N/A	N/A	>2	57 (N/S)	91(N/S)	>10	83 (N/S)	97 (N/S)

Abbreviations: DM96, CellaVision DM96 automated image analysis system; DM9600, CellaVision DM9600 digital cell morphology system; N/A, not applicable; N/S, not stated; RBC, red blood cell.

The detection of conditions associated with the presence of teardrop RBCs in the peripheral blood, including myelofibrosis, myelodysplastic syndrome, and bone marrow involvement by carcinoma, is of significant clinical importance. In a letter to the editor by Egelé et al^26^ from the Albert Schweitzer Hospital in the Netherlands, the results of a local pilot study involving 56 peripheral blood smears (46 containing teardrop cells and 10 controls) was reported. Using a CellaVision (model unspecified) system with Advanced RBC Application software, the overall preclassification sensitivity of the system was reported as 100% for the detection of teardrop cells, with a specificity of 45%. A subset of 10 positive samples was run 10 consecutive times to assess reproducibility, yielding a variation coefficient less than 0.3%. Overall, the authors concluded that digital microscopy systems may well fulfill a future role in the automated screening of blood films for teardrop cells.

Given the clinical importance of the detection of microangiopathic hemolytic anemia, additional work has been completed, with a focus on the ability of the Advanced RBC Application software to detect and classify schistocytes. Hervent et al^27^ examined the blood films of 76 patients (40 controls and 36 abnormal cases) using the CellaVision DM96 system and manual microscopy. The cutoff for the Advanced RBC Application software categorization was optimized by analysis of the ROC curve, yielding a schistocyte cutoff value of 24/1000 RBCs. This cutoff resulted in a sensitivity of 77.8% and a specificity of 93.1% for the detection of a “pathological” quantity of schistocytes. Park et al,^23^ using a cutoff of 5/1000 RBCs, reported a sensitivity of 98.5% (95% CI 92.0%-100%) and a specificity of 58.3% (95% CI, 50.2%-66.2%). The authors noted the software’s difficulty in differentiating acanthocytes from schistocytes. It should be noted that in the 2021 update of the 2012 International Council for Standardization in Hematology recommendations for identification, diagnostic value, and quantitation of schistocytes, the suggested reference range was less than 1% for adults.^28^

Horn et al^25^ from Calgary Laboratory Services in Canada, in a study of 198 smears from a combination of acute-care and community environments, compared the sensitivity and specificity of conventional microscopy with the CellaVision DM96 system in the detection of several clinically significant RBC abnormalities. Red blood cell rouleaux is associated with paraproteinemia and may indicate the presence of an underlying malignant disorder (eg, myeloma or B-cell non-Hodgkin lymphoma).^29^ The sensitivity and specificity of the CellaVision system for the detection of rouleaux was 56% and 97%, respectively. The CellaVision system was insensitive to the presence of RBC agglutination, with a sensitivity of 33% and an associated specificity of 99%. With regard to the presence of Howell-Jolly bodies, a finding in anatomical or functional asplenia, the sensitivity to the detection of any amount was 84%, with a specificity of 94%.^25,29^ This finding is supported by later work by Criel and colleagues,^24^ who reported a similar sensitivity of 83.3% and a specificity of 71.3%.

### Assessment of platelets

The capacity of the CellaVision system to provide an estimation of platelet count is a developing area of interest. Gao et al^30^ from the University of Calgary, Canada, compared the performance of the CellaVision DM96 system with conventional microscopy and their automated hematology analyzers (Coulter LH 780 or Unicel DxH 800 systems [Beckman Coulter]) in the platelet enumeration of 127 peripheral blood samples. The DM96 system demonstrated excellent correlation with both manual estimation (*R*^2^ = 0.94) and the automated analyzers’ estimation (*R*^2^ = 0.92). In a series of 150 blood smears with thrombocytopenia and a “platelet interference flag” on the automated analyzer (HORIBA Yumizen H2500), the PLT-I, PLT-O, and CellaVision DM1200 analyzer methods of platelet enumeration were compared. Correlation between the PLT-O and CellaVision DM1200 methods was high (*r* = 0.809). A subset of 20 samples was compared with the international reference method, with a mean difference in platelet count of 5.72%.^31^

The detection of platelet clumps is an essential component of ensuring quality in full blood count results. Gulati et al^32^ examined 102 blood smears with platelets clumps detected on manual microscopy and assessed the ability of the CellaVision DM96 system to accurately detect their presence. The maximum sensitivity reported was 82.8% and required the user to examine the entirety of the WBC and platelet displays. This limitation of the inability to reliably detect platelet clumping had been noted previously by Billard and colleagues^22^ in the pediatric population but not formally quantified.

## APPLICATIONS OF CELLAVISION SYSTMS IN THE MODERN LABORATORY

### The CellaVision platform in telepathology

Telepathology broadly encompasses the use of a variety of technologies in the practice of pathology across geographically separated locations. The practice has its beginnings in the desire to lend the expertise present in resource-rich areas of the globe to rural or resource-restricted environments.^33^ The CellaVision system is an example of static telepathology that involves the capturing, storage, and transmission of simple static digital images for remote review. The system has been implemented in multisite health services to facilitate the timely diagnosis of blood film abnormalities, without the delay associated with the physical transportation of the sample to the pathologist.^8,34^

Multisite health services must balance the costs and workflow challenges of operating across geographically separated areas. These challenges include the costs associated with the purchase and use of multiple analyzers, the distribution and oversight of trained clinical-laboratory scientists, and the transport and storage of sample material.

#### Correlative performance

Stephens et al from the Department of Pathology at the University of California explored the utility of implementing the CellaVision system for use in remote sites. The study involved the processing of 206 blood samples with a variety of common abnormalities, including thrombocytopenia, monocytosis, and increased immature granulocytes. The samples were processed in an “expensive” hematology analyzer (Sysmex XE5000) and separately on a less expensive hematology analyzer (Sysmex pocH-100i) paired with a CellaVision DM96 system. The Sysmex pocH-100i analyzer provided the nondifferential parameters, with the CellaVision DM96 system providing the differential parameters and blood film images. In a review of the results of the nondifferential parameters, the coefficient of determination was above 0.98 for all parameters. Incorporation of the nucleated RBC count from the CellaVision DM96 system to correct the WBC count of the Sysmex pocH-100i analyzer further increased agreement. The differential counts were compared with a reference method (after classification, following DM96 image review). The Sysmex XE500 analyzer demonstrated good correlation (*R* > 0.8) to the reference method for neutrophil, lymphocyte, eosinophil, and nucleated RBC counts. The CellaVision DM96 preclassification demonstrated numerically superior correlation with the reference method compared with the Sysmex XE500 for basophils (*R*^2^ = 0.625 vs 0.129) and immature granulocytes.

#### Cost-effectiveness

In the financial model proposed, the primary costs of each workflow arrangement were associated with staffing and smear production and review of abnormal results. In the estimations provided over a 5-year period, including upfront costs, the predicted expense of using a complex hematology analyzer setup at a remote site was $2 017 371 vs a simple analyzer system with a CellaVision DM96 at $1 563 316.^35^ The authors concluded that although choices regarding the best hematology laboratory configurations for multisite pathology networks must be individualized, in certain centers with low to moderate daily testing volumes, the use of hematology analysis configurations that include a CellaVision system may lead to decreased costs and potentially improved overall service.

#### Investigational applications

In an exploratory study by Garcia and colleagues^36^ at the University of Pittsburgh Medical Center, patients’ CellaVision images were stored digitally for 6 months and linked to the patients’ electronic health records. This storage allowed clinical hematologists to undertake an online review of patients’ WBC morphology through the hospital’s electronic health record system. The authors noted that the clinicians responded positively to this greater access but highlighted important limitations, including the timeliness of image availability and the limited number of patients for whom the service was available.

### User experience

Paul Panigiris^37^ from SA Pathology presented local experience of implementing the CellaVision DM9600 system into practice at the Public Pathology Australia Quad State National Forum in 2022. Before the introduction of the technology, it was noted that there were difficulties meeting the demands of training and maintaining competency in the area of morphology across a large and geographically dispersed workforce. Early challenges of introducing the new system included staff hesitancy, especially regarding concerns that the CellaVision system scanned only a portion of the slide; information technology constraints, including limited storage capacity; and meeting accreditation standards, including the National Pathology Accreditation Advisory Council requirements for specimen storage. Because implementation of the program has yielded several benefits to the service, including doubling the number of morphologists who can be trained per year, improved workload distribution across sites, and the development of an internal proficiency program accessible across the 17 laboratories. The author concluded that introduction of the system has laid the groundwork for the development of a statewide virtual morphology network to provide integrated service across the entirety of South Australia.

Cory R. Lundgren^38^ from the University of Kansas Medical Center reported the center’s experience of introducing CellaVision Advanced RBC Application software–guided differentials and slide reviews at the Kansas City VA Medical Center in Kansas City, Missouri, a large tertiary-care hospital. The laboratory collected data prospectively using a preintervention and postintervention quasiexperimental design that compared 2 workflows, each over a 3-month period. The preintervention workflow included a Sysmex XN-3000 hematology analyzer and an optical microscope for manual slide review; the postintervention workflow included a Sysmex XN-3000 analyzer, a Sysmex DI-60 digital cell morphology system, and the CellaVision peripheral blood application and Advanced RBC Application software. Of the samples the analyzer flagged for manual differential count, the author described a substantial increase in the proportion of films reported to contain blast cells following technologist review (5.6% vs 20%, *P* < .001). The author hypothesized that this finding may be secondary to an increase in identifying blast cells on blood films with a low or very low blast counts (≤1%). During the study period, a high preclassification false-positive rate for schistocytes (91.7%) was observed, raising the concern that the workflow may contribute to schistocyte misidentification and potentially unnecessary investigation. Overall, the author noted several potential benefits of the CellaVision workflow, including the opportunity for enhanced education and lookback potential, but highlighted the substantial practical challenges in introducing the technology into everyday practice.

Vanvranken et al^39^ from Wexner Medical Center at The Ohio State University, Columbus, Ohio, conducted a real-world user experience survey on the use of the CellaVision DM96 system in the United States and Canada for peripheral blood examination. The survey was sent to 81 laboratories and had a 46% response rate. Using a 5-point Likert average, the top 3 highest-rated benefits of the system were reduced eye strain, reduced interobserver variability, and improved training using digital images for rare and abnormal disorders. The top 3 highest-rated concerns were limitations on performing platelet estimation and RBC morphology evaluation as well as concern that new employees were not gaining skills in manual microscopy if exclusively trained on the CellaVision platform.

Ongoing proficiency assessment of all laboratory staff involved in the morphologic assessment of blood films is a key component of maintaining quality within the hematology laboratory. CellaVision Proficiency Software is a cloud-based software platform that facilitates proficiency assessment through the online presentation of CellaVision slides to individual users, coupled with data analytics and reporting tools. Ingalls et al^40^ from the Department of Pathology at the Health Sciences Centre Nova Scotia published the results of their 6-year experience with the platform. Selected normal and morphologically abnormal slides were selected from within the health network and scanned using either the CellaVision DM96 or DM9600 system. A regimen for grading and assessing responses was established by the medical director, and medical laboratory technologists were encouraged to participate in the program as part of routine education and training. The authors reported several advantages of the system compared with the previous glass slide–based program, including the ability to store and revisit slides, exposure to a wider variety of patient populations, and the ability to review uncommon morphologic findings of emerging interest. Overall, the authors characterized the experience as “very positive,” concluding that the platform was effective as a proficiency assessment program across the geographically dispersed health network.

## EMERGING APPLICATIONS OF THE CELLAVISION SYSTEMS

### Applications of CellaVision systems in parasitology

There have been increasing efforts to exploit the advantages the CellaVision platform offers in the diagnosis of parasitic infections. It should be noted that the application of the CellaVision platform to parasitology remains experimental and not approved by the US Food and Drug Administration (FDA). Within the context of malaria, light microscopy remains the gold standard for diagnosis, despite being a labor-intensive process. Hence, continual developments have been made in alternative technologies, including molecular and fluorescent microscopy techniques, to improve diagnostic accuracy and turnaround time.^41^

The team at the Children’s Medical Centre–Parkland Health and Hospital System in Texas explored the use of the CellaVision DM96 analyzer with the remote viewer software in the diagnosis of *Plasmodium* and *Babesia* species infection on peripheral blood smears. The study compared the identification rates of parasitemia between technologists using light microscopy and the CellaVision DM96 system with remote viewer software. In total, 281 slides were analyzed (130 positive and 151 negative controls). The parasite detection rate was 81% for the optical microscopy group and 73% for the CellaVision remote viewer group, with a Cohen κ coefficient of 0.36. The sensitivity of the CellaVision remote viewer group reached 100% at parasitemia levels above 2.5%.^42^ Attempts have been made to explore whether additional automation and analysis may be useful with the repurposing of the CellaVision Advanced RBC Application technology to us AI to identify infected samples. The Department of Laboratory Medicine at Korea University College of Medicine led a study in which a total of 220 slides were analyzed (84 *Plasmodium vivax*, 14 *P falciparum*, and 122 negative controls). The identification rates of experts using optical microscopy was compared with the CellaVision DM96 system using the Advanced RBC Application. The sensitivity of automatic detection by CellaVision software was only 23.5%, with a substantial proportion of positive samples classified as Howel-Jolly bodies.^43^ A smaller Belgian study using a similar methodology comparing a total of 40 slides (21 *P falciparum*, 3 *P malariae*, 4 *P ovale*, 3 *P vivax*, and 9 negative controls) yielded a slightly higher sensitivity of 72%. There are several possible reasons for this wide discrepancy, including a difference in the median levels of parasitemia and a higher proportion of *P falciparum* in the Belgian study.^44^

Data are limited regarding the utility of the CellaVision system in the detection of other clinically significant intracellular parasites. With regard to human granulocytic anaplasmosis, a case report published by Zhang et al^45^ from Penn State College of Medicine demonstrated the capacity of the CellaVision system to visualize the intracytoplasmic morulae of the *Anaplasma phagocytophilum* bacterium within neutrophils. Cases of anaplasmosis were included within the CellaVision–based morphology proficiency program developed by Ingalls et al,^40^ with an individual case pass rate of 99%. M. Brandon Allen et al^46^ published a single case of *Ehrlichia ewingii* infection, with demonstration of an intracytoplasmic morula in a circulating band form, using the CellaVision platform.

### Applications of the CellaVision platform in body fluid analysis

In addition to the analysis of blood films, the CellaVision platform has been granted FDA approval for the analysis of body fluids. These fluids can pose unique challenges, including variable cellularity, protein content, and commonly the presence of both hematopoietic and nonhematopoietic cells. Ivady et al from the University of Debrecen in Hungary investigated the utility of the CellaVision DM96 system compared with manual light microscopy in the analysis of ascitic fluid and cerebrospinal fluid (CSF). Two cytospin preparations of each of 80 body fluid samples (40 ascitic fluid and 40 CSF) collected over a 2-year period were prepared and stained with May-Grünwald Giemsa stain. Two separate experienced investigators using either light microscopy or the CellaVision DM96 system then classified the cells into the following categories: neutrophils, eosinophils, lymphocytes, monocytes, unidentified, other or non-WBC. Samples were additionally analyzed using flow cytometry and 2 automated cell counters (Sysmex XN-1000 and XN-2000). Using a linear regression model, the CellaVision platform demonstrated a high degree of agreement with the light microscopy method for both ascitic fluid (*r* = 0.984) and CSF (*r* = 0.969).^47^ In earlier work by Riedl et al^48^ with the Department of Clinical Chemistry and Hematology at Albert Schweitzer Hospital, similar concordance was demonstrated using the CellaVision DM96 system to analyze 177 samples (62 ascitic fluid, 100 CSF, 15 abdominal ambulatory peritoneal dialysis) compared with light microscopy. The study reported the preclassification accuracy as 90% for CSF and 83% for the aggregate of other body fluids. Following human review of the CellaVision findings, there was a high rate of agreement between light microscopy and the CellaVision system for CSF (*r* = 0.92-0.99) and other bodily fluids (*r* = 0.83-0.98).

An exploratory study led by Dr Laurits Frøssing^49^ from Bispebjerg Hospital in Copenhagen, Denmark, investigated the comparative accuracy of light microscopy and the CellaVision DM96 system in the identification of eosinophilia in sputum samples. Fifty sputum samples from patients with asthma were prepared using standard protocols with albumin 20% to prevent cell smudging. There was modest preclassification agreement between the methods for the identification of sputum eosinophilia (κ = 0.61) and poor agreement in the identification of sputum neutrophilia (κ = 0.23). One case has been reported in the literature of the use of the CellaVision DM96 system in the diagnosis of T-lineage ALL presenting with a pleural effusion.^50^ Further work is required to assess the accuracy and applicability of the CellaVision platform in these settings.

### Applications of CellaVision systems in building AI models

Efforts are ongoing to develop AI models with the capability to automatically classify blood cells based on their morphologic characteristics. Modern models frequently employ convolutional neural networks, which require large volumes of high-quality training data. Hence, repositories from CellaVision, with its capability to capture, store, and transmit blood smear images, have been useful for this purpose. Andrea Acevedo and colleagues^51^ from Hospital Clinic of Barcelona have generated one of the largest publicly available repositories of CellaVision images, with more than 17 000 professionally annotated images of cells captured in routine clinical practice. The dataset, in combination with images from the CellaVision website, has been used to compare AI models and validate specific data-extraction elements required for accurate classification, including cell segmentation, nucleus segmentation, and texture.^52-54^ These technologies remain in the developmental stages, and large datasets such as those generated by the CellaVision system are poised to play a substantial role in the creation of future models.

## CONCLUSION

The integration of digital microscopy systems such as the CellaVision into hematology laboratories represents a notable advancement in blood film analysis. These systems offer numerous benefits, including rapid morphologic analysis, remote review capabilities, and improved occupational health and safety. The CellaVision technology has demonstrated high accuracy in preclassifying neutrophils and lymphocytes, but challenges remain in the classification of basophils and immature granulocytes, including blast cells. The Advanced RBC Application has shown varying levels of sensitivity and specificity for different RBC types, with several critical limitations, including the ability to detect RBC agglutination and classify schistocytes.

The application of the CellaVision platform to telepathology further enhances its utility, particularly in multisite laboratory services. By enabling the timely diagnosis of blood film abnormalities without the need for physical transportation of samples, the CellaVision systems address key challenges associated with geographically separated laboratory operations. Emerging applications of the CellaVision platform in parasitology show promise, particularly in the diagnosis of malarial infection, but data are limited to small case series. The sensitivity of automatic detection remains variable, and ongoing research and technological advancements may enhance the platform’s diagnostic accuracy and utility in parasitology.

In the context of ongoing deficiencies in several critical clinical scenarios, human input and oversight remain paramount to ensure best outcomes for patients. It is critical that efforts to clinically validate the technology across a broad range of scenarios continue if the CellaVision systems are to see widespread adoption in routine clinical practice. Despite their current limitations, however, there is little doubt that the CellaVision and similar digital microscope systems are poised to play an increasingly important role in the modern hematology laboratory, with the overarching goal of providing accurate and timely diagnosis of blood film abnormalities.
